# Plasma Levels of Cytokines (IL-10, IFN-γ and TNF-α) in Multidrug Resistant Tuberculosis and Drug Responsive Tuberculosis Patients in Ghana

**DOI:** 10.3390/diseases7010002

**Published:** 2018-12-23

**Authors:** Anthony Basingnaa, Samuel Antwi-Baffour, Dinah Obenewaa Nkansah, Emmanuel Afutu, Enid Owusu

**Affiliations:** 1 Department of Medical Laboratory Sciences, School of Biomedical and Allied Health Sciences, University of Ghana, Accra, Ghana; tbasingnaa@yahoo.com (A.B.); s.antwi-baffour@chs.edu.gh (S.A.-B.); donkansah@ug.edu.gh (D.O.N.); 2 Ghana Health Service, PMB, Ministries, Accra, Ghana; 3 Department of Medical Microbiology, School of Biomedical and Allied Health Sciences, College of Health Sciences, University of Ghana, Accra, Ghana; eafutu@ug.edu.gh

**Keywords:** cytokines, DS-TB, MDR-TB, *Mycobacterium tuberculosis*, tuberculosis

## Abstract

The emergence of multidrug-resistant tuberculosis (MDR–TB) and more recently, extensively drug-resistant (XDR) TB has intensified the need for studies aimed at identifying factors associated with TB drug resistance. This study determined the differences in plasma concentrations of pro-inflammatory (IFN-γ and TNF-α) and anti-inflammatory (IL-10) cytokines in MDR-TB and drug-susceptible (DS) TB patients, in addition to some socio-economic factors. Plasma levels of IL-10, IFN-γ and TNF-α were measured in 83 participants (comprising 49 MDR-TB and 34 DS-TB patients) using sandwich ELISA. Levels of the three cytokines were elevated in MDR-TB patients compared to DS-TB patients. The mean level of IL-10 (7.8 ± 3.61 *ρ*g/mL) measured in MDR-TB cases was relatively higher than those of TNF-α and IFN-γ, and statistically significant (*p* = 0.0022) when compared to the level of IL-10 (4.8 ± 4.94 *ρ*g/mL) in the DS-TB cases. There were statistically significant associations between MDR-TB and factors such as education level (*X*^2^ = 9.895, *p* = 0.043), employment status (*X*^2^ = 19.404, *p* = 0.001) and alcoholism (*X*^2^ = 3.971, *p* = 0.046). This study adds to the knowledge that IFN-γ, TNF-α and IL-10 play a role in the host response to *Mycobacterium tuberculosis* (*MTB*). Alcohol intake can be considered as an important MDR-TB risk factor.

## 1. Introduction

Tuberculosis (TB) remains one of the world’s deadliest communicable diseases [1]. Despite the availability of drugs to treat and control the disease, certain factors including patients’ non-compliance, poor nutrition, and co-infection with HIV/AIDS has resulted in increased burden of TB infection, especially in resource-limited countries within sub-Saharan Africa and Asia [1,2]. 

In 2017, the World Health Organization (WHO) reported that 490,000 million cases of multidrug-resistant TB (MDR-TB) emerged in 2016 and an additional 110,000 cases were susceptible to isoniazid but resistant to rifampicin (RR-TB), the most effective first-line anti-TB drug [3]. The advent of multidrug-resistant cases pose a disproportionate threat to the global prospects of TB control, and frustrates diagnosis and treatment [4,5]. The regions most affected by MDR-TB cases are the former Soviet Union (Eastern European countries) and the Russian Federation, where at least one-third of TB cases presenting for treatment have MDR-TB [6,7]. In sub-Saharan Africa, South Africa is reported to have the highest number of MDR-TB cases [8]. Population rates of MDR-TB cases in a number of countries across the world have increased over time [9,10]. In Ghana, MDR-TB (resistance to at least isoniazid and rifampin, plus resistance to several drugs, excluding combined resistance to isoniazid and rifampin) among TB patients has been found to be 8.7% [11]. 

Cytokines have been identified to play multiple roles in the immune and pathological responses in TB. Interleukin-10 (IL-10), interferon gamma (IFN-γ) and tumour necrosis factor (TNF-α) play very important roles in anti-TB cell-mediated immunity, in both MDR-TB and drug-susceptible TB (DS-TB) patients [12,13,14]. TNF-α has been identified to play various roles in the immune and pathological responses in TB by preventing the reactivation of persistent tuberculosis, modulating the pulmonic expression of specific immunologic factors and limiting the pathological response of the host [14]. IFN-γ boosts antigen presentation, leading to the recruitment of CD4+ T-lymphocytes and cytotoxic T-lymphocytes [12], while IL-10 in response to *Mycobacterium tuberculosis* (*MTB*), may down-regulate the immune response and limit tissue injury. However, overexpression of these cytokines may have a negative impact on the capacity to control infection [13]. Effective disease management strategies must therefore consider both drug treatment and host immunity. This strategic approach can be successful if studies are conducted on the immune markers involved and devise ways of enhancing their function to maximize effective response, especially in MDR-TB. This study therefore determined the levels of cytokines (IL-10, IFN-γ and TNF-α) that are linked with the ability of *MTB* to evade the host’s immune system and mediate long-term infections in the lungs, leading to chronic tuberculosis.

## 2. Materials and Methods

### 2.1. Study Design, Subjects and Sample Collection 

From March to September 2016, participants were enrolled from six Regional TB treatment (Directly Observe Therapy Short course—DOTS) centres, including the Chest Clinic of the Korle-Bu Teaching Hospital (KBTH), which is the national referral clinic [15]. The study population comprised two groups of participants.

Group 1 was made up of MDR-TB patients. These were TB patients who showed resistance to the first-line anti-tuberculosis drugs, especially rifampicin (R) and isoniazid (H), while being on treatment for a 6-month period. These groups of patients were monitored regularly to prevent the possibility of re-infection or reactivation after successful case treatment. MDR-TB patients were confirmed by the Gene X-pert TB analysis as well as culture/drug susceptibility testing (DST) [16]. These patients were sputum-positive and had typical signs and symptoms of TB. It was however beyond the scope of the current study to establish whether infection was caused by a TB sensitive strain which could have developed resistance in the course of treatment, resulting from initial treatment regimen, or if the patients were infected with a strain that was already drug-resistant. Four individuals identified to be HIV-positive were excluded from the study. In addition, 49 HIV-negative blood specimens, confirmed by the First Response HIV 1-2 kits (Premier Medical Corporation Ltd., Kachigam, India) and HIV “ECLIA” (electrochemiluminescence immunoassay, Roche Diagnostics GmbH, Penzberg, Germany) were used for the study.

Group 2 consisted of 34 TB patients confirmed to be HIV-negative who had been on TB treatment for 6 months and had become smear-negative for TB bacilli prior to study enrolment. It was ensured that this group had also been treated with the first-line anti-tuberculosis drugs just like those in group 1, in order to rule out the possibility of potential differential treatments being a confounding factor that could affect the cytokine profiles observed in both groups. The smear negativity for TB bacilli in group 2 was confirmed by sputum smear microscopy. 

National guidelines for pulmonary tuberculosis therapy (involving two months of intensive phase of rifampicin (150 mg), isoniazid (75 mg), pyrazinamide (400 mg) and ethambutol hydrochloride (275 mg) and 4 months of the continuation phase with only rifampicin and isoniazid) were used in the treatment of the two groups. 

None of the study participants in both groups were experiencing any co-morbidities at the time of sample collection. Patients in both groups were however not investigated for extra-pulmonary TB, hence they were all considered as pulmonary TB cases. 

All 83 patients (comprising 49 MDR-TB patients and 34 DS-TB patients) gave their informed consent for inclusion before they participated in the study. The study was conducted in accordance with the Declaration of Helsinki, and the protocol was approved by the Ethics and Protocol Review committee of the School of Biomedical and Allied Health Sciences, College of Health Sciences, University of Ghana (Approval number: SBAHS/10080166/AA/MLS/2015-2016). In addition, permission was obtained from the Ghana National Tuberculosis Control Programme before the study was carried out.

A structured questionnaire on age, gender, education level, marital status, location and risks factors including smoking and alcohol consumption were administered in order to obtain participants’ data. This was done before blood samples were collected from the participants. 

### 2.2. Plasma Level Measurement of Cytokines (TNF-α, IFN-γ and IL-10) 

Four millilitres (4 mL) of blood samples form the study participants were centrifuged (Beckman TJ-6, New York, NY, USA) at 3000× *g* for 5 min, and plasma supernatants were pipetted into Eppendorf tubes (Brinkman Instrument Inc., Westbury, NY, USA). The measurement of the three cytokines was done by using Sandwich ELISA. A DuoSet®ELISA commercial kit (R&D Systems, Minneapolis, MA, USA) is among the ELISA pair sets that are built on sandwich ELISA, and therefore was used for the current study. Each of the serum samples were tested three times in the sandwich ELISA assay, and the average was recorded. 

Briefly, NuncMaxiSorp 96-well plates (Fisher Scientific, Loughborough, UK) were coated separately with capture antibody in duplicates for TNF-α, IFN-γ and IL-10 at working concentrations of 4 μg/mL, 4 μg/mL and 2 μg/mL respectively. This was done in filtered phosphate-buffered saline (PBS) at a volume of 50 μL/well and sealed with a plate sealer, after which the set up was incubated overnight at room temperature. 

Plates for each cytokine were washed thrice with 0.05% Tween 20 (Sigma-Aldrich, St. Louis, MO, USA) in PBS to remove unbound capture antigen, and blocked with 200 μL/well of 1% bovine serum album (BSA) (Sigma-Aldrich, St. Louis, MO, USA) in PBS (filtered) for 1 h and then washed thrice again. Then, 50 μL/well of sample plasmas and cytokine standards (diluted 2-fold from 1000 *ρ*g/mL to 7.80 *ρ*g/mL for TNF-α and IFN-γ while IL-10 was 2000 *ρ*g/mL to 15.7 *ρ*g/mL) were added in duplicates and incubated at room temperature for 2 h. 

Bound antibodies for each cytokine were detected by their detection antibody and diluted to working concentrations of 400 ηg/mL for TNF-α and 75 ηg/mL for IL-10 in diluent buffer 1% BSA in PBS while IFN-γ was 200 ηg/mL in 0.05% Tween 20 and 0.1% BSA in Tris buffer saline diluent for 2 h at room temperature. Affinities for each cytokine antibody were detected with streptavidin horseradish peroxidase (HRP) conjugate (diluted forty times in each diluent buffer) at room temperature for 20 min in the dark. Colour development was detected by adding 2,2′-azino-bis (3-ethylbenzothiazoline-6-sulphonic acid) (ABTS) liquid substrate (Sigma-Aldrich, St. Louis, MO, USA) at room temperature for 20 min in the dark, and the reaction was stopped by adding 25 μL/well of 2 N sulphuric acid. The optical density (OD) was measured with an Absorbance Microplate Reader ELx 808 (BioTek Instruments, Vermont, VT, USA) at 450 nm, with wavelength correction set at 540 nm. After each incubation step, contents within the microtiter plate wells were decanted, and wells of the microtiter plates were washed with 150 μL/well of 0.05% Tween 20 in PBS, three times.

### 2.3. Statistical Analysis

Data obtained were entered and stored into Microsoft Excel 2010 (Microsoft Corp., Redmond, Washington, WA, USA), and statistical analysis was performed using STATA statistical software version 12 (STATA Corp, Texas, TX, USA). Association between social factors in MDR-TB was analysed using Chi-square (*X^2^*), and odds ratios by linear regression. All plasma concentration results were represented as means ± standard deviation (SD). A *p*-value < 0.05 was considered statistically significant.

## 3. Results 

A total of eighty-three pulmonary TB cases were used in the study. These included forty-nine MDR-TB and thirty-four DS-TB patients. The MDR-TB cases were made up of 34 (69.4%) males and 15 (30.6%) females, while DS-TB cases were made up of 23 (67.7%) males and 11 (32.3%) females (Table 1). Most of the MDR-TB cases had ages greater than 20 years. The mean age of MDR-TB cases was 44.0 years, while that of the DS-TB cases was 36.0 years. The ages of MDR-TB cases was not statistically different from that of DS-TB cases (*X*^2^ = 2.571, *p* = 0.111). Additionally, there was no statistically significant association between MDR-TB and factors such as gender (*X*^2^ = 0.028, *p* = 0.866) and marital status (*X*^2^ = 1.126, *p* = 0.570). 

The majority of participants in the MDR-TB (81.6%) and DS-TB (94.1%) cases were non-smokers, but no significant association existed between MDR-TB and patient smoking status (*X*^2^ = 2.721, *p* = 0.099). Meanwhile, there were statistically significant associations between MDR-TB patients and factors such as educational level (*X*^2^ = 9.895, *p* = 0.043), employment status (*X*^2^ = 19.404, *p* = 0.001) and alcoholism (*X*^2^ = 3.971, *p* = 0.046), as shown in Table 1. The odds ratio of MDR-TB cases having tertiary level of education was less by a magnitude of 0.0007 over that of DS-TB cases that did not have tertiary education, and this was statistically significant (*p* = 0.016). Likewise, the odds ratio of MDR-TB cases drinking alcohol was statistically significant (*p* = 0.015), and this was also greater by a magnitude of 113.07 compared to DS-TB cases. 

### Plasma Levels of Pro- (IFN-γ and TNF-α) and Anti-Inflammatory Cytokines (IL-10) in MDR-TB and DS-TB Cases

**Figure 1:**
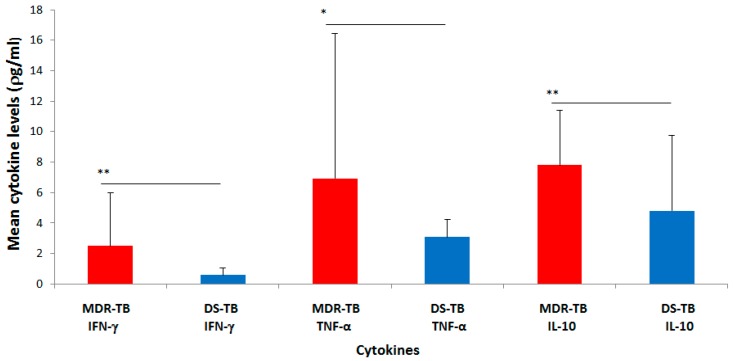
Mean plasma concentrations (*ρ*g/mL) of IFN-γ, TNF-α and IL-10 in MDR-TB and DS-TB patients. Graph of data represents mean ± standard deviation, where ** *p* < 0.01 and * *p* < 0.05.

The mean concentration of IFN-γ measured in the plasma of MDR-TB cases was found to be 2.5 ± 3.51 *ρ*g/mL (mean ± standard deviation (SD)) (Figure 1). This was statistically significant and higher (*p* = 0.0019) than the mean plasma concentration found in the DS-TB cases (0.6 ± 0.44 *ρ*g/mL). Likewise, the mean concentration of TNF-α measured in the plasma of MDR-TB cases (6.9 ± 9.53 *ρ*g/mL) was significantly higher (*p* = 0.0213) than the level in DS-TB cases (3.1 ± 1.15 *ρ*g/mL). Furthermore, the mean concentration of the anti-inflammatory cytokine (IL-10) measured in MDR-TB cases was observed to be 7.8 ± 3.61 *ρ*g/mL. This plasma level of IL-10 (7.8 ± 3.61 *ρ*g/mL) was relatively higher than that of the pro-inflammatory cytokines (IFN-γ and TNF-α: 2.5 ± 3.51 *ρ*g/mL and 6.9 ± 9.53 *ρ*g/mL, respectively) and significantly higher (*p* = 0.0022) when compared to the level of IL-10 in the DS-TB cases (4.8 ± 4.94 *ρ*g/mL). 

The relatively higher mean concentration of the anti-inflammatory cytokine (IL-10) compared to that of the pro-inflammatory cytokines (IFN-γ and TNF-α) observed for the MDR-TB cases was similar to what was observed for the DS-TB cases (Figure 1).

## 4. Discussion

### 4.1. Relationship between MDR-TB and Socio-Demographic Information

The mean age recorded for MDR-TB and DS-TB patients in this study was similar to other studies conducted in Taiwan and Bolivia [17,18], where the mean ages were 43.30 and 33.90 years, respectively. In Ghana, retirement age is pegged at 60 years, while voluntary retirement is also placed at 55 years [19]. Therefore, it is very important to note that the mean age of 44 years recorded in this study is still within the productive age group, and thus, individuals suffering from MDR-TB can be incapacitated by the disease and might not be able to do much to contribute economically. This can result in a negative economic impact to society and the country as a whole. The finding that the majority of the MDR-TB patients were males is in accordance with other studies which revealed that there are generally more males than females suffering from TB and MDR-TB worldwide [17,18,20]. Studies have found that TB is gender-sensitive. The burden of the disease is greater on women due to stigma and societal settings than on men, even though more men suffer from TB worldwide than women [20,21,22]. In a study by Rao [23] that looked at TB and patient gender, it was observed that the male-to-female ratio in pulmonary tuberculosis patients was 2:1, and that this was in agreement with other reports [24,25]. 

Generally, TB infection has a direct effect on the well-being of the individual, and those who attain MDR-TB status become compromised in finding a job or continuing with their duties if they are employed [26]. Poverty has been found to be strongly associated with TB and MDR-TB [26]. According to a review by Lienhardt et al. [27], poverty and the tubercle bacillus create a second vicious circle. Poor people living in rural areas, suffering from hunger and crowded into close non-hygienic places are easily victimized in an environment where TB is easily spread [27]. Meanwhile, in the current study, MDR-TB was found more in individuals living in the city, urban and suburban areas. Due to the abundance of drug stores in these areas compared to the rural areas, there is the greater tendency for such people to self-medicate. Additionally, the busy lifestyle of people living in the city and other urban areas could result in TB patients not completing their treatment courses. These situations encourage the emergence of drug-resistant TB strains [28], and could explain the link between residency and MDR-TB observed in this study. Similar to findings from the current study, Bhatias et al. [29] reported on the link between unemployment, non-adherence of TB patients to TB medication and the development of drug resistance. The current study also found alcohol intake to be a risk factor associated with MDR-TB patients, and this finding agrees with a report by Zetola et al. [30] in Botswana. Abuse of alcohol is common in sub-Saharan Africa, where TB, MDR-TB and HIV are very prevalent [31,32]. This occurrence has been associated with medical non-adherence, and has led to poor health status and overall worse clinical outcomes of such people [31,32]. Patients diagnosed with TB have been observed to have higher alcohol intake than non-TB individuals [33,34]. 

### 4.2. Plasma Concentration of the Cytokines (IL-10, IFN-γ and TNF-α)

Chronic immune activation, which might be because of exposure to a high load of environmental antigens, has mostly characterized the immune profile of people living in sub-Saharan Africa [35,36,37]. Such exposure has been observed to impair the host’s immune response to *M. tuberculosis* and HIV [35], which are widespread in sub-Saharan Africa [38]. Infection with intracellular parasites such as *Mycobacterium tuberculosis* is known to induce Th1 immune response [38]. The protective immunity against the TB pathogen is said to be mediated by cytokines such as IFN-γ, TNF-α, IL-12, IL-6 and IL-18 during the initial stage of infection [39]. 

IFN-γ has been identified to be significant for the function and maturation of multiple immune cells [40]. It stimulates macrophages to produce TNF-α, which is an essential component of the innate defence mechanism of the host against pathogenic challenge [41]. IL-10 plays an important role in suppressing macrophage and dendritic cell (DC) function, which helps control and initiate the immune responses [42]. TNF-α contributes to the pathogenesis of tuberculosis due to its role in the formation and maintenance of granulomas [43]. TNF-α is considered a necessity in the removal of bacteria in inflammatory lesions, and hence it has been found to be one of the key cytokines in controlling MTB infection [44,45]. Therefore, it was not surprising that elevated plasma levels of TNF-α were found in MDR-TB participants compared to the DS-TB participants. This correlated with a Chinese study that showed elevated levels of TNF-α in TB patients compared to controls [46]. Several studies have reported increased levels of TNF-α in the serum of TB patients [47,48,49]. TNF-α is a factor both in the protection against tuberculosis and in immunopathology. The high levels of TNF-α in MDR-TB patients in the current study may be due to a marked tissue necrosis during the disease occurrence that led to progressive TB and eventually MDR. This might have resulted in the release of TNF-α into circulation and eventually contributed to systemic indicators of TB, such as fever and cachexia.

The role of IFN-γ as the main macrophage-activator (Th1 cytokine) has been clearly established in animal models infected with *M. tuberculosis* [50]. Intracellular mycobacteria are destroyed due to IFN-γ action on macrophages [41,51]. IFN-γ has been shown to stimulate macrophages, leading to the following: production of TNF-α, oxygen free radicals and nitric oxide; increase in the surface display of MHC antigens and Fc receptors; increase in the intracellular concentration of some antibiotics; and decrease in lysosomal pH [41,51,52]. The significantly higher plasma levels of IFN-γ in MDR-TB compared to DS-TB observed in this study may be because more IFN-γ action on macrophages will be needed to destroy intracellular mycobacteria that show multi-resistance to anti-TB drugs. 

IL-10, which is a suppressor T cell or T-regulatory cytokine, is known to play a critical role during chronic and latent stages of pulmonary TB [53]. The IL-10 production is said to be elevated during the infection, promoting reactivation of TB [54]. The excessive production of this cytokine usually results in failure to control the infection, and this could account for why IL-10 was elevated in the MDR-TB participants. Increased production of IL-10 in patients with active disease including MDR-TB has been reported in Turkey [54]. This high IL-10 production in MDR-TB might also indicate suppression of the immune response, leading to an inadequate balance of pro- and anti-inflammatory cytokines. 

This study has some limitations which have been pointed out. Patients in the two groups were not investigated for extra-pulmonary TB, hence they were all considered as pulmonary TB cases, and this was considered a limitation in the current study. Additionally, sputum smear microscopy was the only method used to confirm smear negativity for TB bacilli. Finally, the sample size obtained for smokers in MDR-TB and DS-TB was quite low, and might have masked an association.

## 5. Conclusions

Tuberculosis is highly endemic in Ghana and yet, to the best of our knowledge, this study is the first to directly compare plasma levels of cytokines (IL-10, IFN-γ and TNF-α) in MDR-TB and DS-TB patients in the country. The levels of both pro- (IFN-γ, TNF-α) and anti-inflammatory (IL-10) cytokines were observed to be significantly higher in MDR-TB patients compare DS-TB patients. The findings of this study emphasize the potential roles of IFN-γ, TNF-α and IL-10 in the host response to MTB during drug resistance development. This study also showed a statistically significant association between MDR-TB and factors such as education level, employment status and alcohol intake. Alcohol intake can be considered as a very important risk factor due to the alcoholism attitude of most Ghanaians, in both urban and rural areas.

## Figures and Tables

**Table 1:** Socio-economic and risk factor information of the study participants (N = 83).

Characteristics	MDR-TB Cases, n (%)	DS-TB Cases, n (%)	*p*-Value	Chi-Square (*X*^2^)
**Gender**				
Male	34 (69.4)	23 (67.7)	0.866	0.028
Female	15 (30.6)	11 (32.3)		
**Age (years)**				
18–23	8 (16.3)	10 (29.4)	0.111	2.571
24–29	5 (10.2)	5 (14.7)		
30–35	9 (18.4)	6 (17.7)		
36–41	13 (26.5)	9 (26.5)		
42–47	12 (24.5)	3 (8.8)		
48–53	2 (4.1)	1 (2.9)		
**Marital status**				
Married	26 (53.1)	22 (64.7)	0.570	1.126
Separated	13 (26.5)	7 (20.6)		
Divorced	10 (20.4)	5 (14.7)		
**Residency**				
City	15 (30.6)	0 (0.0)	0.005	12.946
Urban	15 (30.6)	14 (41.2)		
Suburban	17 (34.7)	17 (50.0)		
Rural	2 (4.1)	3 (8.8)		
**Education Level**				
Tertiary	1 (2.0)	6 (17.6)	0.042	9.895
High school	11 (22.5)	10 (29.5)		
J.S.S.	3 (6.1)	4 (11.7)		
Middle school	4 (8.2)	1 (2.9)		
Elementary school	30 (61.2)	13 (38.3)		
**Employment Status**				
Employed	5 (10.2)	11 (32.4)	0.001	19.404
Self-employed	10 (20.4)	15 (44.1)		
Farmer	6 (12.2)	0 (0.0)		
Student	4 (8.2)	5 (14.7)		
Unemployed	23 (46.9)	3 (8.8)		
**Alcohol Intake**				
Alcohol drinkers	22 (44.9)	8 (23.5)	0.046	3.971
Non-Alcohol drinkers	27 (55.1)	26 (76.5)		
**Smoking Status**				
Smokers	9 (18.4)	2 (5.9)	0.099	2.721
Non-smokers	40 (81.6)	32 (94.1)		

J.S.S represents junior secondary school, n represents number, % represents percentage.
